# Dr. W. T. Law

**Published:** 1910-09-17

**Authors:** 


The death is announced of Dr. W. T. Law at the age of sixty-five. Dr. Law was educated at Guy's and Edinburgh, taking his M.B. degree in 1872 and his M.D. in 1874, and admitted a F.R.C.S. England, in 1875. Dr. Law had held appointments at the *Dreadnought* Hospital, Greenwich, the Royal Infirmary, Edinburgh, and the Chorlton Hospital, Manchester, and he had written considerably on medical subjects.

